# Factors Influencing Consumption Intention of Insect-Fed Fish among Italian Respondents

**DOI:** 10.3390/foods12173301

**Published:** 2023-09-02

**Authors:** Luca Mulazzani, Brunella Arru, Luca Camanzi, Roberto Furesi, Giulio Malorgio, Pietro Pulina, Fabio A. Madau

**Affiliations:** 1 Department of Agricultural and Food Sciences, Alma Mater Studiorum, Università di Bologna, Via Zamboni, 33, 40126 Bologna, Italy; luca.mulazzani@unibo.it (L.M.); luca.camanzi@unibo.it (L.C.); giulio.malorgio@unibo.it (G.M.); 2 Department of Agricultural Sciences, University of Sassari, Via Enrico de Nicola 1, 07100 Sassari, Italy; rfuresi@uiss.it (R.F.); ppulina@uniss.it (P.P.); famadu@uniss.it (F.A.M.); 3 Interdepartmental Center for Industrial Agrofood Research (CIRI-AGRO), Alma Mater Studiorum, Università di Bologna, Via Zamboni, 33, 40126 Bologna, Italy; 4 National Biodiversity Future Centre, University of Palermo, Piazza Marina, 61, 90133 Palermo, Italy

**Keywords:** theory of planned behavior, moral attitude, sustainability consciousness, country of origin, price sensitivity, Italian consumers

## Abstract

The rise in the world’s demand for fish is increasingly met by aquaculture. However, this sector still shows various criticalities in terms of sustainability of practices, first and foremost, that of feed availability. Nowadays, the use of insect meal represents one of the potential sustainable solutions, but consumption intention of fish fed with insect meal and the factors affecting it have not yet been adequately understood. This study investigates 318 Italian consumers’ intentions to buy fish fed with insect meal using an extended version of the Theory of Planned Behavior, including consumers’ moral attitude and sustainability consciousness as additional constructs. The results of structural equation models show that consumers’ high sustainability consciousness (6.16 on a scale from 1 to 7) does not influence their consumption intention of this product. Also, the two moderating variables involved in the model, i.e., the country of origin and price sensitivity, do not significantly affect consumers’ intentions. Since the analysis demonstrates that, for consumers, insect meal-fed fish conforms to their moral principles and a significant positive attitude toward this practice it could be argued that fish fed with insect meal can match the demand from consumers who feel responsible for their consumer behavior. Although the limited area of investigations and the high education of interviewed do not allow for generalizing of the results, this paper provides pivotal food for thought for companies, policymakers, and academics responding to previous research calls on understanding the role of some constructs of consumption intention and highlighting the levers on which to act to foster the consumption intention of insect-fed fish.

## 1. Introduction

The increase in world population, the awareness of the significant impact of eating habits on personal health, as well as the mental and physical health benefits of eating fish will result in an estimated increase in the demand for fish of 28 million tons by 2030 compared to 2018 (+18%) [1,2,3]. This demand is increasingly met by aquaculture, which is expected to provide most of the fish in the future considering the normative constraints applied to fisheries worldwide [1]. However, aquaculture still shows various criticalities in terms of technology, production, and, more generally, the sustainability of farming practices. One of the main critical points is that fish farming is still deeply rooted in the use of feed derived from limited resources, namely fish meal (FM) [4,5].

Currently, FM is considered the best protein source for fish feed because it boasts an optimal protein and amino composition [6]. Nevertheless, this property collides with its unsustainability due to competition between human and animal industries, increasing production costs and ecological challenges [7,8]. The search for sustainably sourced food and feed protein requires reconsidering protein procured for human and animal needs. This is even more true for the aquaculture sector, which is the fastest growing food industry in the world [1] and which urgently requires different protein feed sources to become more sustainable.

The above aquaculture sector’s huge need can be met by using insects for feed [9]. Recently, European Union legislation (EU Reg. 217/893) has allowed the introduction of feed derived from certain insects in animal diets. However, the food insects and food production sector face numerous regulatory, technological, and cultural challenges [10,11]. This opening of the European Community is consistent with Goal 12 of Agenda 2030 for Sustainable Development aimed at ensuring sustainable consumption and production, and the Farm to Fork strategy [12], which strives to make food systems fair, healthy, and environmentally friendly. In fact, the opportunity of adopting insect meal (IM) in aquaculture—rather than other alternatives to FM, such as the criticized soy [5,6,13,14]—stems from IM’s many properties. IM has been praised for its distinctive features: it has a very similar amounts of protein, minerals, and vitamins to FM, in addition to high energy, fat, and fiber content [15]; it is a part of the natural diet of freshwater and marine fish, especially in the juvenile stage [5,6,16]; it can be produced locally by smallholder farmers [17]; and it has a low ecological impact [15] in accordance with Goal 14 of the Sustainable Development Agenda. Given these properties, fish fed with IM can fall into the sustainable food category [18,19] and allow far-reaching changes in the aquaculture sector [20].

Culture plays a significant role in determining the acceptability of insect consumption, so people who live in areas of Asia, Africa, and Central America view insect consumption positively and practice entomophagy [21]. The introduction of insects in diets is typically regarded with disgust, diffidence, and skepticism in European countries due to huge phycological, social, cultural barriers, and demographic issues [11,22,23,24,25,26]. In effect, even though farmed animals are not fed insects whole and people do not ingest insects directly, cultural and psychological barriers may not necessarily be overcome [11]. The cultural context influence in the acceptance of insect-based foods results from the significant impact of food culture and ethnic background on consumer food choices [25,27]. Moral standards of culture determine the disgust towards food and what is or is not edible [28,29]. Other factors that merit attentions are religion, ethnicity and acculturation [26]. In Western countries, although some early market niches have developed, insects still remain often considered sources of contamination and disease vectors [28,30]. On the contrary, there has been a long history of using insects as a natural resource in China, and there are many practices and experiences that describe the use of insects (as well as food as a medicine), especially in rural areas [31]. In China, over 300 insect species are taxonomically classified as edible, and their patent applications have increased rapidly, proving that edible insect development and utilization is ongoing [32]. This has prompted increased attention to investigation on the toxicity of edible insects in the context of the modern food industry [33]. Specifically, the new mandatory standard of procedures for toxicological assessment of food issued by the National Health and Family Planning Commission of P. R. China is a particularly important part of the national food safety standards and contains improved scientificity and universality [33].

In this context, IM has recently gained significant interest as a viable fish feed alternative, and there have been several requests to expand the research on consumers’ opinions, preferences, intentions and the factors affecting this, and acceptability regarding IM use in aquaculture [7,11,34,35,36], where the results to date are overall optimistic [7,26,37,38,39,40,41]. It was noted that providing consumers with information about the benefits and safety of insects as feed can increase their willingness to consume animals fed with insects, yet consumers know little about these topics [35,40]. In light of these primordial studies that suggest consumers have different approaches to “insects as food” and “insects as feed”, and given that the two themes can be analyzed from different angles in different disciplines [42], it is reasonable to assume that the two concepts are not contiguous. This study focuses exclusively on the consumer approach to insects as feed.

Given this background, the consumer plays a key role in transitioning towards a sustainable food system and implementing sustainable aquaculture [43,44]. The successful introduction of IM in the aquaculture sector depends on the consumers’ intentions to eat fish fed with IM [35], making it essential to better understand the factors that determine this food choice. The analysis of factors behind the “consumption intention” of fish fed with IM appears particularly suitable to understand how to foster this food innovation. However, despite increasing public and academic interest in the subject, several questions are still waiting to be answered [45]. In particular, only recently has some academic research focused on consumer acceptance of IM and the factors affecting it, as well as on consumer awareness and attitudes toward sustainable aquaculture and willingness to pay for fish fed with IM [34,36,39,40], resulting in persisting low understanding of the topic.

Nowadays, although most consumers do not care about the type of feed when buying fish, and their level of knowledge about insect-based feed is low, two considerations are necessary. On the one hand, the Farm to Fork strategy [12] aims to make consumers more aware of what they eat, and consumers’ concerns about food quality are extremely high [46]. On the other hand, technologies such as the blockchain greatly increase transparency, allow monitoring of the product flow in the fishery supply chains, ensure food safety, and prevent fraudulent activities [47]. Faced with the availability of much greater information for consumers in the near future, it is important to understand which levers to intervene on to obtain acceptance of IM for fish feeding as soon as possible. It is also of the utmost importance for producers to exploit the best opportunities on the market [48] in light of the need to increase the internal production of fish and strengthen the aquaculture industry [49] in a sustainable way.

### Theoretical Background of the Study

The TPB is one of the most widely notable and referred to socio-psychological guidelines for understanding, predicting, and explaining human behavior [50]. It has also been frequently applied in the past to predict many food-related behaviors in regard to green and sustainable food, genetically modified food, risky food, and innovative products including functional food, or even healthy and dietary eating [28,49,51,52,53,54,55,56]. The TPB’s success also lies in being a useful framework for designing interventions to induce behavior changes [57]. The TPB states that the intention to perform the behaviors is the most proximal predictor for the behavior itself and is a function of underlying motivational variables: “attitude” (ATT), “subjective norms” (SUN), and “perceived behavioral control” (PBC).

A favorable or unfavorable “attitude” towards a particular behavior derives from beliefs about the likely consequences of the behavior practiced. Beliefs about the presence of factors that may enable or obstruct the behavior’s performance trigger the “perceived behavioral control”, which refers to a person’s perception of the ease or difficulty of performing a specific behavior. The “subjective norms” refers to the belief about the expectations of approval or disapproval of other people to perform a specific behavior. Commonly, the greater the “attitude”, “perceived behavioral control”, and “subjective norms” are, the stronger one’s intention to perform the behavior is.

It is important to mention that the “subjective norms” arise from the full set of “descriptive normative beliefs” and “injunctive norms” (INJ) [50,58]. The first relates to observing social referents’ behaviors and revealing our beliefs about what others have done or are doing. The second concerns the peer group’s perceived moral rules about certain behaviors and assists a person in determining what acceptable and unacceptable social behaviors are. Due to the novelty of investigating food products and the related consumer behavior, we only considered “injunctive norms” [49].

In light of the above, a first group of hypotheses was formulated:

**H1a.**  *“Attitude” positively affects “consumption intention” of fish fed with insect meal.*

**H1b.**  *“Perceived behavioral control” positively affects “consumption intention” of fish fed with insect meal.*

**H1c.**  *“Injunctive norms” positively affect “consumption intention” of fish fed with insect meal.*

Ajzen [59] argued that a modified TPB model that encompasses additional pivotal constructs in a specific context contributes to understanding the model’s theoretical mechanism and enhances the prediction power for individuals’ intentions and behaviors in such contexts. Since the investigated context refers to sustainable foods, two other constructs appear relevant. The TPB model has been criticized for not considering moral influences on behaviors [60]. Ajzen [59] suggested including “moral norms” MON (also defined as personal norms and moral obligations) among the other three predicted constructs of the behavioral intention model. The “moral norms” refer to a person’s belief that acting in a certain way is intrinsically right or wrong, notwithstanding personal or social consequences [61]. The “moral norms” have proven to be a significant addition to the TPB model in the food context while being valuable in capturing sustainable food consumption behaviors [51,62,63,64,65].

The “moral norms” include two aspects that act oppositely on the overall construct but are nevertheless related to it: the norm and the attitude [51]. The first refers mainly to negative feelings (guilt or obligation), which arise when a person’s moral values are violated and act as motivators of behavior intended to avoid the negative consequence. The second relates to viable positive outcomes of fulfilling one’s moral values. According to Arvola et al. [62] and Olsen et al. [66], we refer to a “moral attitude” (MOA) as a situation in which a person is aware that the wellbeing of others relies on his or her actions and feels responsible for the actions and their consequences. These persons try to fulfill their moral obligation, but if they cannot (because there is no opportunity to do so) they are less likely to suffer negative consequences; they do not feel a personal moral value violation. Positive consequences to the self arise from positive self-enhancing feelings of doing what is believed to be right, evoking emotions such as pride or self-satisfaction. Other previous studies in the food context adopted the same viewpoint [51,62,66,67]. In this study, we want to investigate the possible positive consequences of fulfilling one’s moral values (in this case, consuming sustainable fish), so we focus on the influence of “moral attitude” on the intention of consuming fish fed with IM.

The role of “sustainability consciousness” (SUC) in consumer behavior—which refers to the experience or awareness of sustainability phenomena [68] and influences behavioral intentions [69]—is controversial. According to Bangsa and Schlegelmilch [70], “sustainability consciousness” does not always translate into actual behavior, and there is still a need to understand to what extent “sustainability consciousness ”affects “consumption intention” deeply. On the contrary, Balderjahn et al. [71] consider “sustainability consciousness” an important antecedent in determining sustainable purchasing behaviors. According to Balderjahn et al. [72], sustainable consumption consciousness is a state of concern “to consume in a way that enhances the environmental, social and economic aspects of quality of life”. In this vein, Sheth et al. [73] and Lim [74] introduce the concept of conscious consumption, that is, more conscious behaviors due to a sense of caring for nature, community, and the self. Consumers who are aware of the importance of sustainability are more likely to account for this construct in their consuming and purchasing decisions in a bid to make sustainable choices. Previous studies which focused on environmental awareness consider it an important factor for improving the acceptance of eating insects [49,75,76], and in respect of the TPB model, it has a favorable impact on intention towards buying environmentally sustainable products [77]. In this study, we assume that “sustainability consciousness” is a crucial antecedent in determining an individual’s intention to consume fish fed with IM and should be considered an additional component of the extended TPB model.

Based on the above, the following second group of hypotheses was developed:

**H2a.**  *“Moral attitude” positively affects “consumption intention” of fish fed with insect meal.*

**H2b.**  *“Sustainability consciousness” positively affects “consumption intention” of fish fed with insect meal.*

According to Ajzen [50], some “background factors” could indirectly influence intentions and behaviors given their effects on predictors. Previous studies evidence the prediction power of the “country of origin” (COO) and “price sensitivity” (PRS) on buying decision, and their importance in explaining customer consumption behaviors, regarding both fish and, sustainable products on the whole [11,25,78,79,80].

The “country of origin” is an extrinsic cue for evaluating products and is linked to the image, stereotype, and reputation that producers and consumers assign to products of a specific country based on variables such as national traits, political circumstances, and economy, history and traditions [81]. Numerous studies have shown that “country of origin” is one of the most important aspects of consumer purchase intention and choice [38,78,82,83]. The “country of origin” has been highlighted as a signal of product quality, safety, and freshness [84,85,86], as well as having significant positive effects on “attitude”, “perceived behavioral control”, and SN [87,88,89]. When analyzing the ethical and unethical food consumption among Finnish, Danish, and Italian students, Mäkiniemi et al. found that the students consider food produced very close to them as ethical. According to Fleșeriu et al. [90,91], for the consumer, buying national rather than international products can represent an obligation to support local communities. In positive terms, it is possible to assert that consumption of national food can generate the consumer’s feeling of doing the right thing because he or she is contributing to the wellbeing of local businesses.

In addition, previous literature contributions showed that the “country of origin” could be an important factor, specifically when purchasing seafood [83,92]. In fact, consumers may rely on this information as a cue that either affects their perception or helps them to make informed choices (e.g., due to different national regulations) about the safety, quality and sustainability of the seafood they purchase and consume. Hence, it is reasonable to hypothesize that the “country of origin” could mediate the relationship between “attitude”, “perceived behavioral control”, “injunctive norms”, “moral attitude”, “sustainability consciousness”, and the “consumption intention” of fish fed with insect meal.

Insect feed acceptance is strongly affected by price [38,40,78]. “Price sensitivity” has been defined as “the extent of consciousness and reaction displayed by consumers when finding differences among the prices of given products or services” [93] and even “the extent to which a customer accepts a rise in price for a specific product in terms of economic and psychological gains” [94]. Overall, consumers who feel an ethical responsibility toward society and the environment demonstrate this through their consumption behavior [95]. In the context of eco-friendly/green and sustainable products, previous studies describe “price sensitivity” as a “willingness to pay more” for products with characteristics of sustainable production [96,97,98,99]. Consumers’ willingness to pay extra for ethically produced goods has been demonstrated, particularly for those perceived as enhancing producers’ livelihoods and for sustainable food [100,101,102,103]. In the field of a sustainable alternative to FM research, Llagostera et al. [7] found that Spanish consumers were willing to pay a premium for fish fed with IM rather than with fish meal. In the same context, when investigating the use of insect-based foods as an alternative source of protein, Vartiainen et al. [25] found that price and convenience significantly affect the intention to consume insect-based foods.

Thus, based on the above, the following third group of hypotheses was developed:

**H3a.**  *“Country of origin” of fish positively mediates the relationship between “attitude”, “perceived behavioral control”, “injunctive norms”, “moral attitude”, and “sustainability consciousness” and “consumption intention” of fish fed with insect meal.*

**H3b.**  *“Price sensitivity” positively mediates the relationship between “attitude”, “perceived behavioral control”, “injunctive norms”, “moral attitude”, and “sustainability consciousness” and “consumption intention” of fish fed with insect meal.*

**Figure 1:**
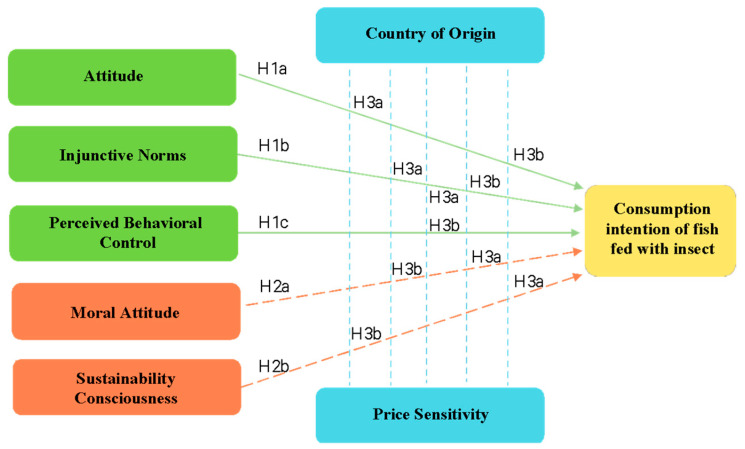
Hypothesis research model. The TPB’s original variables (attitude, injunctive norms and perceived behavioral control) (H1) as well as the additional constructs (Moral Norms and Sustainability Consciousness) (H2) positively affects consumption intention of fish fed with IM. The Country of Origin and Price Sensitivity act as positive mediators in the relationships between the original variables and additional constructs of the TPB model (H3).

The research model and hypotheses are shown in Figure 1.

The present paper contributes to filling gaps in literature by assessing consumers’ intentions to eat fish fed with IM. Specifically, this paper aims to evaluate “consumption intention” of fish fed with IM and to provide insight into the factors that affect that intention. The Theory of Planned Behavior (TPB) [104] was chosen as the theoretical framework of the study since it has been proven appropriate for understanding sustainable and ethical consumer behaviors concerning food [65,105,106,107,108]. Although previous studies used the TPB to investigate the “consumption intention” of food products containing insect meal i.e., [109], consumers’ actual sustainable behavior in the assessment of consumers’ willingness to incorporate insects into diets i.e., [i.e., 38], to our knowledge, no previous study has used the TPB to investigate the “consumption intention” of fish fed with IM. In particular, an extended version of the TPB model was employed to include additional constructs considered necessary in a sustainable food context since they directly (“moral norm” and “sustainability awareness”) and indirectly (“country of origin” indication and “price sensitivity”) influence the intentions and behaviors of consumption regarding fish and sustainable products [11,25,63,64,69,78,79,80].

The analysis was carried out among Italian consumers. This choice arises from the high importance of fish in the Italian diet, consumed at least a few times a week by 62.1% of Italians [110]. Moreover, in EU-27, Italy ranked first in household nominal expenditure on fishery and aquaculture products in 2021 and experienced the most significant expenditure increase between 2020 and 2021 (7% equal to more than EUR 880 million) [111]. In such circumstances, (i) the sustainability of seafood production and consumption is an important global issue, (ii) fish stocks in the Mediterranean are constantly decreasing and the amount of fish caught cannot increase [112], and (iii) increasing seafood consumption and production poses a number of environmental challenges that require adequate measures to meet them [113]. Investigating what levels can be used to promote environmentally sustainable aquaculture appears to be an important field of research, with many theoretical and practical implications.

## 2. Materials and Methods

### 2.1. Sampling and Data Collection

This study employed an online survey created with Google Forms that was disseminated via social networks and was in the field for two months. Given the purpose of the research, the questionnaire was disseminated in Italian to collect information only from Italian people. A convenience sampling approach with no stratifications for inherent characteristics (e.g., social, demographic, economic) was employed to collect data, and no respondents received incentives for participating in this study. We chose this approach because interviewing only people interested in the subject of the investigation minimizes the risk of protest votes. Although the sample may not be representative of the population, the choice came from the desire to direct the survey to fish consumers and/or to those genuinely interested in the subject. The non-probability sampling, such as voluntary opt-in web panels, is a sampling technique which is very popular in market research [114] and studies consumer preferences to auto-select the sample based on the real interest of people in consuming a particular product.

By the closing date for survey responses, 320 answers were recorded, and 318 valid questionnaires were retrieved, all provided by Italian people. Since the questionnaire comprises 26 questions, the final sample meets the prior condition of at least 10 cases per parameter [115].

### 2.2. Measures of Constructs

With the purpose of testing the hypotheses, measurement items were developed to adequately represent the eight research constructs, which are “consumption intention” (INT) of fish fed with IM (INJ, PBC, ATT, MOA, SUC, COO, and PRS). All items employed were previously tested by other scholars and adopted in their original form or in a slightly modified version. Since the sample is made up of Italians, it should be noted that when consumers make their purchase choices, they can choose to buy national or foreign fish. The importance of the “country of origin” variable fits into this context. An overview of the measurement system of the model is provided in Table A1 of the Appendix A.

### 2.3. Questionnaire Design

The first step of our questionnaire construction concerned the translation into Italian of all items based on the English literature and back translation to ensure the translation’s quality and meaningfulness [116]. Afterward, the questionnaire was pre-tested to verify the absence of potential errors or misunderstandings concerning its general design and the items used. The questionnaire was composed of several parts also aimed at answering other research questions presented in other research papers [117]. Specifically, the first and second parts of the questionnaire are shared with other research activities, while the third is specific to this study. After being welcomed to the study and ensured of the reliable and anonymous use of the data provided, the participants received a brief explanation of fish feeding problems in the aquaculture sector and some information on the economic, ecological, social, health, and nutritional information of insect feed. No information was provided about product traits or appearance.

In the second part of the survey, respondents were asked about their socio-cultural characteristics: individual information on gender, age, education, and job occupation. The third covered questions concerning respondents’ concerns about all constructs of our model (Appendix A). All indicator variables were assessed using 7-point Likert scales (i.e., 1 = strongly disagree; 7 = strongly agree) due to their potential offer of higher reliability and validity compared to shorter scales [118]. The English translation of the questionnaire can be retrieved from https://forms.office.com/e/YvAp8B6qeh (accessed on 28 August 2023).

### 2.4. Measures

The research model was analyzed and interpreted in a sequence of two stages: the assessment of the adequacy of the measurement model, which is useful for testing the reliability and validity of the scales employed, and the evaluation of the structural model.

The assessment of the measurement model involves several steps [119].

First, a Factor Analysis (FA) with Principal Component Analysis and Varimax rotation was employed to detect the questionnaire’s validity and test the constructs’ unidimensional structure. The validity was determined using Factor Loadings (FL), which should be greater than 0.50 [120]. The dataset was further evaluated using Kaiser-Meyer-Olkin (KMO) [a measure of sampling adequacy to assess the appropriateness of using factor analysis on the data set, with a cut-off value of 0.5 – [121] and Bartlett’s test of sphericity (a test for the null hypothesis that the correlation matrix has an identity matrix) to test the scale’s structure validity. Furthermore, descriptive statistics were provided. Specifically, we estimated the mean scores (M) and the standard deviation values (SD) of the constructs and the Pearson correlation coefficients (r), which allowed us to investigate the existence of a positive relationship between constructs and describe the strength of this relationship [122].

Second, to determine the reliability, i.e., the degree of the internal coherence of a set of indicators, Cronbach’s α, for which acceptable values are 0.50 [123,124] and the Composite Reliability (CR) for each construct (threshold limit value of 0.70) [125] was investigated. The Average Variance Extracted (AVE) for each construct (cut-off value 0.50) [126] demonstrated a convergent validity, and hence research model constructs that were adequate were applied.

As Hair et al. [125] suggested, a structural model allows for capturing the linear regression effects of the endogenous construct upon one another.

The following structural equation states the structure of the full model.

(1) $$\begin{array}{cc} INT= & \beta_{1}ATT+\beta_{2}INJ+\beta_{3}PBC+\beta_{4}SUC+\beta_{5}MOA+\beta_{6}ATT\times COO+\beta_{7}INJ\times COO+\\ & \beta_{8}PBC\times COO+\beta_{9}SUC\times COO+\beta_{10}MOA\times COO+\beta_{11}COO+\varepsilon \end{array}$$

(2) $$\begin{array}{cc} INT= & \beta_{1}ATT+\beta_{2}INJ+\beta_{3}PBC+\beta_{4}SUC+\beta_{5}MOA+\beta_{6}ATT\times PRS+\beta_{7}INJ\times PRS+\\ & \beta_{8}PBC\times PRS+\beta_{9}SUC\times PRS+\beta_{10}MOA\times PRS+\beta_{11}PRS+\varepsilon \; \end{array}$$

*INT*: Consumption intention; *ATT*: Attitude; *INJ*: Injunctive norm; *PBC*: Perceived behavioral control; *SUC*: Sustainability consciousness; *MOA*: Moral attitude; *COO*: Country of origin; *PRS*: Price sensitivity.

The above equations showed that all basic (*ATT*; *INJ*, PBC) and additional (*SUC*; *MOA*) constructs are predictors of “consumption intention” (*INT*) and that the singular relationships between such constructs and the “consumption intention” are a function of the “country of origin” and “price sensitivity” constructs. In other words, the “country of origin” and “price sensitivity” individually interact with all basic and additional constructs to edit the five relationships between the latter and the “consumption intention”.

Equations (1) and (2) include the coefficients of *β*_1_, *β*_2_, *β*_3_, *β*_4_, *β*_5_, and *β*_11_ to verify the direct effect of all constructs, including those of the moderators [127] on “consumption intention”, and the coefficients of *β*_6_, *β*_7_, *β*_8_, *β*_9_, *β*_10_, to consider the moderating effects of “country of origin” and “price sensitivity” on the relationships between basic and additional constructs and “consumption intention”.

The constructs’ effects and their interactions on “consumption intention” were evaluated using a moderated hierarchical regression analysis applying a structural equation model (SEM) [128]. Specifically, entering independent variables and interactions in two blocks gave rise to two nested models (for each moderator construct), the basic and moderation models. The first indicated the effect of original and additional constructs and the controlled effect of “country of origin” (and “price sensitivity”) on “consumption intention”. The second included five additional moderator effects.

According to Kline [115], to assess whether the structural equation of the four models fit well with the data, we used the comparative fit index (CFI), the Tucker–Lewis index (TLI), and the root mean square error of approximation (RMSEA) with 90% confidence. An acceptable fit based on these indexes is considered RMSEA ≤ 0.08 and CFI and TFI ≥ 0.9. The coefficient of determination (R2) informs on the explained variance of the endogenous variables (INT). The BM SPSS AMOS 26.0 (IBM, Armonk, NY, USA) was used.

## 3. Results

### 3.1. Sample Profile

The demographic information showed that respondents were 51.72% male and 48.28% female; most (42.95%) were aged between 40 and 49 years, 38.24% had a university degree, and 29.47% had a postgraduate degree. The data are reported in Table 1.

### 3.2. Measurement Model Adequacy Assessment

The FA results on all constructs confirmed their unidimensional structure. Factor loading analysis revealed support for reliability validity of the eight constructs as these were above 0.50 [125] (see Table 2). Sample adequacy was proven as all constructs showed a KMO greater than the threshold limit value of 0.5 [121] and Bartlett’s test of 0.00.

The Pearson correlation coefficient (r) was employed to investigate the existence of a positive linear relationship among all constructs and to describe the strength and direction of this relationship. The results of the correlation analysis are all statistically significant (*p* < 0.01) and positive. The intention was found to be moderately associated with “perceived behavioral control”, strongly with “price sensitivity” and “injunctive norms”, and very strongly associated with “attitude”. In contrast, a low association was detected between “consumption intention”, “sustainability consciousness”, and “country of origin”. In effect, the last construct showed a weak association with all other constructs. Also, although “sustainability consciousness” has the highest mean, it revealed a weak association with other constructs except for “perceived behavioral control” (see Table 3).

Internal consistency analysis of the measured constructs showed high reliability for all constructs. Findings showed that Cronbach’s α was between 0.78–0.93, except for “perceived behavioral control” and “sustainability consciousness”, which were 0.50 and 0.62, respectively, thus acceptable values [123,124]. The CR revealed an adequate convergent validity, showing values between 0.74–0.96, exceeding the cut-off value (0.70) suggested by Fornell and Larcker [126], and the AVE from each construct exceeded 0.5, thus providing evidence of discriminant validity (Ibidem).

### 3.3. Structural Equation Model Estimation

A set of tests was run to estimate the suitability of the expanded Ajzen model with respect to the alternative restricted models. A generalized likelihood-ratio test (*GLRT*) procedure was adopted, and the statistic associated with it is reported as follows:

(3) $$GLRT=-2ln=-2\;\left(ln\frac{L\left(H_{0}\right)}{L(H_{1})}\right)=-2\;ln\;L\left(H_{0}\right)\;–\;ln\;L\left(H_{1}\right)\;$$

where *L*(*H*_1_) and *L*(*H*_0_) are the log-likelihood values of the expanded and the restricted models, respectively. The statistical test λ has approximately a chi-square or a mixed-square distribution with a number of degrees of freedom that corresponds to the number of restrictions (parameters assumed to be zero in the *L*(*H*_0_) null hypothesis). If the value of λ is significantly lower than the corresponding critical value (for α = 0.05 significance level), the null hypothesis can be rejected, and the preferred model would not involve these variables. The results are reported in Table 4.

The first test concerned the exclusion of both “moral attitude” and “sustainability consciousness” variables. The starting (null) hypothesis (*MOA* = *SUC* = 0) was compared with the adopted hypothesis (extended model). The null hypothesis was rejected, meaning the extended model is preferable to the Ajzen Original Model.

The second and third tests were based on the null hypothesis, in which a single additional variable was removed. We found that the model without “sustainability consciousness” cannot be significantly rejected; therefore, the preferred model would include only the “moral attitude” variable (Table 5).

Furthermore, we also estimated the models which were suitable for the data using the GFI, CFI, and RMSA fit statistics. The results confirmed that the Ajzen Original Model with “moral attitude” is the one that best suits the data (Table 4).

Therefore, adopting the Ajzen model with the “moral attitude” variable as the basic one, we estimated the effects of the moderating variables. Results are reported in Table 6 and Table 7.

The assessment of the four-model fit of Table 6 revealed that data fit, according to Kline [115], in only two models: the basic Ajzen Model with “moral attitude” and the basic Ajzen Original Model. The R2 of these models showed that three independent variables (“attitude”, “injunctive norms”, “moral attitude”) are good predictors of the value of the “consumption intention” of fish fed with IM. In particular, “attitude” is the variable with the greatest impact on the “consumption intention” of IM-fed fish. The effect of “perceived behavioral control” is not statistically significant, nor is that of the control variable “country of origin”.

Models with the “price sensitivity” moderator revealed that only the basic Ajzen Model with “moral attitude” fits, according to Kline [115], and results similar to those of the COO-moderated models were found. Specifically, as shown in Table 7, the *p*-values of the “perceived behavioral control” and “price sensitivity” regressors are not relevant to the explanation of the dependent variable.

In sum, as reported in the Table 8, only two hypotheses of the first group were confirmed (i.e., H1a and H1c), while in the second group of hypotheses, only the first one (MOA affects INT) matched our results. When considering the moderating effect of the “country of origin” and the “price sensitivity”, both hypotheses of the third group were rejected. The SEM showed that only the “price sensitivity” has an influence, albeit very small, on consumer intentions.

## 4. Discussion

This study draws upon and contributes to contemporary academic and political debates aimed at making the aquaculture sector more sustainable. In this sense, an effective ally is insect meal, which currently appears to be a valid alternative to fish meal. Among the various players involved in the market, this article focuses on consumers, responding to the request for more research aimed at understanding consumers’ perspective towards the “consumption intention” of fish fed with insect meal [35] and the factors affecting this perception [34,36]. Responding to these appeals is of paramount importance to (i) support producers in formulating appropriate strategies for market development, (ii) help them to promote a market for insect-fed fish, and (iii) enable them to exploit better market opportunities [48,129] to make aquaculture a sustainable sector.

Considering the extensive use of the TPB in analyzing consumer intent in the context of food and also sustainable food. As far as we know, we were the first to investigate constructs that can help predict “consumption intention” of insect meal-fed fish by expanding the analysis to two more constructs, namely “sustainability consciousness” and “moral attitude”, and adding “country of origin” and “price sensitivity” as moderating constructs.

The role of the “sustainability consciousness” is controversial—some research has found that it is an important antecedent of sustainable purchasing behaviors [71] and that it has a positive impact on intentions towards buying environmentally sustainable products [77]. Others highlight that it does not always translate into actual behavior [70]. This paper meets the request of thoroughly investigating its impact on consumption intention, given that consumers’ sustainable product choices are highly context-dependent [70]. In this study, respondents showed a very high level of “sustainability consciousness”, but this is not an antecedent to the “consumption intention” of fish fed with IM. The explanation of this result can be fourfold.

Firstly, respondents may have overestimated their sustainability awareness, which may be unrelated to their true intention [130]. According to van Dam and van Trijp [131], as the importance of sustainability reported by consumers is driven by abstract considerations, it can be less predictive of consuming decisions than more realistic measures. In this regard, our results agree with Grunert et al. [132], according to who high levels of consumer concern for sustainability issues at an abstract level correspond to lower levels of consideration in the concrete context of food choices.

Another possible explanation for this result can be the social desirability effect, which, although some studies attribute a minor role to it i.e., [133], can be a potential confounding variable in the sustainability research field [134]. The social desirability, that is, the interviewee’s tendency to give answers that make him or her look better, can also occur in anonymous online surveys [135].

In contrast to the previous explanations, there is a possibility that respondents indeed care about sustainability. Yet, they are not fully persuaded that insect meal-fed fish is a sustainable product, and the explanation in the questionnaire’s introduction did not entirely convince them.

A final explanation may be that, as suggested by Saidi et al. [136], Italian consumers do not worry about environmental sustainability in choosing fish because they consider the environmental impact of their consumption behavior to be very low.

Concerning the second added construct to TPB, our findings confirm previous studies [51,62,63,64,65] that see the “moral attitude” as a meaningful addition to the TPB increasing the model’s predictive capacity.

As expected, this construct, which refers to personal beliefs about what is morally right and wrong, is strongly correlated with the perceived morals of a person concerning his or her interpersonal networks and the surrounding community and that determine if a behavior is acceptable or unacceptable (“injunctive norms”).

In order to better understand the factors that most influence consumers in choosing fish fed with insect meal, we tested the role of two variables indicated by previous studies as crucial in consumer choices in general and the choice of fish specifically, namely “country of origin” and “price sensitivity”.

The analysis of the “country of origin” effect revealed its low correlation with other constructs of the model. Given this result, its performance in the SEMs is not surprising. Besides not having an indirect moderating effect on the other model constructs, its direct impact on the “consumption intention” of insect meal-fed fish is not statistically significant. Our findings contrast with previous research that infantized its role in “consumption intention” [38,78,82,83] and in the basic constructs of Ayzen’s model [87,88,89].

Two possible explanations can be found in the role of information.

According to Vanhonacker et al. [137], consumers’ perceptions of aquaculture and farmed fishery depend more on emotional considerations rather than on rational ones, and, due to little knowledge or awareness regarding the origin of fish by European consumers, they do not prioritize the “country of origin” of fish as an information cue.

Conversely, the results of Saidi et al. [136] reveal a preference in Italian consumers for local fish but also a significant trust in fishmongers. This data can mean that if the fishmonger explains to the consumer why the fish is insect meal-fed and guarantees the safety and goodness of the fish, the “country of origin” can take a back seat.

Regarding the “price sensitivity” variable, our results differ from previous studies [7,25] which see this construct as a pivotal element to consumer choice in the context of fish consumption.

A partial answer to the low importance of price can be found in the study of Ankamah-Yeboah et al. [78], where one out of three of the interviewees was more concerned about the attributes rather than the price of the fish and did not show a significant preference for fish fed with insect meal, showing their indifference to the feed used. In fact, consumers are currently poorly informed about the feed used to feed fish [78] and a previous study [138] showed that people commonly do not particularly care about the feed the animals consumed.

Our results also clash with those of Saidi et al. [136]. According to them, price is more relevant for Italian seaside residents than inland residents as the former are more interested in other factors (i.e., freshness, availability, and seasonality) and less sensitive to price when the fish satisfies their needs. Indeed, although the questionnaire was addressed to people from all Italian regions, our sample consisted of 251/318 residents on the island of Sardinia.

Despite these possible explanations for the results recorded in the additional and moderator constructs to Ajzen’s original model, the high value of the “attitude” recorded in the various models cannot be overlooked. This finding confirms previous studies that see positive consumer attitudes towards the consumption of insect meal-fed fish, an essential result as “attitude” is crucial in determining consumer acceptance [139,140]. Specifically, positive consumers’ “attitude” is the primary factor determining whether they accept situations that are beneficial to the environment but require effort for them [139]. In fact, a positive attitude can compensate for the effort—i.e., dealing with the uncertainty and risk involved when adopting a new product—needed to adopt a sustainable behavior for the environment [141].

A further relevant result is the non-significance of “perceived behavioral control” in influencing the choice to consume fish fed with IM. The “perceived behavioral control” mirrors the influence of perception of personal capacities and constraints about the target behavior on intentions and the extent of individual control over the performance of the behavior. Also in this case, it could be argued that the consumers’ limited knowledge about the feeds of fish and perhaps also the little interest in the subject, as shown in previous studies, can explain the absence of the role of this antecedent on consumption intention.

Concerning the antecedent “injunctive norms”, due to previous mixed evidence about the influence of people’s perceptions of what those who matter to him or her approve of or expect them to do, this paper responds to the call for research to deepen the impact of injunctive norms on eating behaviors [142]. The detection of a moderate role of the “injunctive norms” confirms previous studies that saw it as a weak predictor of intentions [49]. A relevant element to underline emerged by analyzing the answers to every single item of the construct. The question that obtained the highest score is the one that investigated whether the interviewee would consume fish fed with insect meal if dietary guidelines recommended eating them. Therefore, even if the influence of this construct is limited, the role that institutions can play in reassuring and recommending the use of insect meal in fish feeding appears very important.

The study has some limitations that should be overcome in future research. Although the sample size complies with Kline’s dictates [115], we cannot generalize the data obtained in our research because of the limited area of investigations to only Italy. Moreover, the problem of self-selection should be considered [143], as respondents were free to participate in the survey. Consequently, it is likely that mostly people with an interest in the topic (in both positive and negative terms) participated. In addition, the respondents in this study were highly educated, with implications for our findings that limit the study’s application to the Italian population at large. It would be interesting to extend the questionnaires to other countries. Finally, the “intention-behavior gap” must be mentioned, as an intention does not necessarily mean it will be put into practice.

## 5. Final Remarks and Implications

By showing the variables that affect consumers’ intentions to buy fish fed with insect meal, this work provides food for thought for companies that want to approach this sustainable way of production, indicating which levers to act upon.

Since consumers demonstrate that the consumption of insect meal-fed fish conforms to their moral principles and those they believe to be of other influential persons, and do not care about the difficulty or otherwise of being able to perform this behavior, it could be argued that fish fed with IM can match the demand of consumers who feel responsible for their consuming behavior. This work can be helpful for companies that already use insect meal or are considering using it. With the forthcoming requests from the European community for more information on food production processes, due to the positive attitude shown, such companies need not fear a boycott. The results show the need for a push towards communicating the sustainability of such products and suggest that fishmongers could be important intermediaries.

As a result of our work, several implications can be raised for academics.

Certainly, consumers’ perceptions of the product in terms of sustainability are interesting since the results seem to suggest that consumers may not understand fish fed with IM as sustainable food. In this respect, it is necessary to point out that other studies have shown (i) the importance of price in consumer choices when buying food products that were perceived as containing attributes relevant to sustainability [144,145], and (ii) that this sensitivity is particularly evident for some European fish consumers that buy sustainably produced fish from Europe since they trust the standards applied and feel they are well communicated [103]. Therefore, future research should investigate how to make the consumer understand that the IM-fed fish are a sustainable product and what kind of information makes less informed consumers able to distinguish between conventionally fed fish and IM-fed fish. In this way, consumers could accept a premium price for insect meal-fed fish, offsetting the still high costs of this feed [4].

This work responds to previous research calls, in some cases validating (weak predictor effect of “injunctive norms”) and in others contrasting (non-role of “country of origin”) earlier research that emphasized the role of the analyzed constructs on consumption intention. Future research should deeply investigate the role of “perceived behavioral control”. More specifically, as this construct is superordinate concerning two other antecedents, i.e., perceived self-efficacy and perceived controllability, future research should investigate if one of those can be a lever in the “consumption intention” fish fed with insect meal. Furthermore, future research in which respondents could actually try or buy fish fed with insects would significantly complement our findings.

This work also has pivotal implications for policymakers. European Commission (EC), with its new policies (EU Green Deal with F2F and Biodiversity strategies, Horizon Europe, Next Generation EU), is engaged in making European food the global standard for sustainability. However, nowadays, insect meal is still too expensive [4]. The only way to make this product attractive is to make consumers sensitive to price, highlighting the attributes of sustainability as well as the dietary recommendations that they value, and which lead to the acceptance of greater spending.

## Figures and Tables

**Table 1:** Demographic profile of the sample.

	Total No. = 318	%
Gender		
Male	165	51.7
Female	153	47.9
Age		
18–29	45	14.1
30–39	56	17.5
40–49	136	42.6
50–59	55	17.2
≥60	26	8.1
Education		
Lower middle school	11	3.4
High school	91	28.5
University	122	38.2
Post University	94	29.5
Occupation		
Artisan	6	1.9
Cleric	1	0.3
Desk Job	42	13.2
Entrepreneur	11	3.4
Freelance	61	19.1
Government job	20	6.3
Househusband/housewife	3	0.9
Non-university Teacher	30	9.4
Pensioner	13	4.1
Private employee	40	12.5
Researcher/University Professor	39	12.2
Student	41	12.8
Unemployed	10	3.1
Not answered	2	0.6

**Table 2:** Factor loading.

Constructs and Indicators	Factor Loadings	KMO
Attitude		0.73
ATT1	0.92	
ATT2	0.88	
ATT3	0.94	
Injunctive norms		0.80
INJ1	0.89	
INJ2	0.90	
INJ3	0.91	
INJ4	0.89	
Perceived behavioral control		0.55
PBC1	0.80	
PBC2	0.63	
PBC3	0.67	
Consumption intention		0.72
INT1	0.97	
INT2	0.96	
INT3	0.91	
Country of origin		0.74
COO1	0.93	
COO2	0.96	
COO3	0.92	
Price sensitivity		0.68
PRS1	0.91	
PRS2	0.92	
PRS3	0.57	
PRS4	0.67	
Sustainability consciousness		0.60
SUC1	0.68	
SUC2	0.80	
SUC3	0.87	
Moral attitude		0.76
MOA1	0.94	
MOA2	0.92	
MOA3	0.92	

**Table 3:** Construct means, standard deviations, and correlations.

	M.	S.D.	ATT	INJ	PBC	INT	COO	PRS	SUC	MOA
Attitude	4.52	1.85	1							
Injunctive norms	4.27	1.81	0.873	1						
Perceived behavioral control	4.95	1.22	0.604	0.563	1					
Consumption intention	4.65	1.90	0.919	0.865	0.649	1				
Country of origin	4.67	1.50	0.297	0.332	0.355	0.348	1			
Price sensitivity	4.26	1.33	0.689	0.653	0.550	0.720	0.378	1		
Sustainability consciousness	6.16	1.00	0.392	0.374	0.544	0.446	0.378	0.466	1	
Moral attitude	4.35	1.87	0.793	0.802	0.600	0.831	0.402	0.677	0.396	1

All correlations are significant at a level of 0.01 (2-tailed).

**Table 4:** Testing the theoretical model by SEM with only original and additional constructs.

Variables	Ajzen’s Original Model		Ajzen’s Original Model and MOA		Ajzen’s Original Model and SUC		Extended Ajzen Model	
	Std. β	*p*-Value	Std. β	*p*-Value	Std. β	*p*-Value	Std. β	*p*-Value
ATT	0.75	0.00	0.67	0.00	0.76	0.00	0.68	0.00
INJ	0.32	0.00	0.21	0.00	0.32	0.00	0.21	0.00
PBC	0.07	0.05	0.03	0.38	0.13	0.01	0.08	0.09
SUC					−0.05	0.12	−0.05	0.16
MOA			0.23	0.00			0.22	0.00
R2	0.98		0.98			0.98	0.98	
Correct R2	0.98		0.98			0.98	0.98	
*p*-value(F)	0.00		0.00			0.00	0.00	
Log-likelihood	−343.72		−325.70		−342.26		−324.48	
x2	231.91		313.21		458.26		549.80	
CFI	0.96		0.96		0.93		0.93	
TLI	0.95		0.95		0.91		0.91	
RMSEA	0.09		0.08		0.11		0.09	

**Table 5:** Tests of hypotheses for the extended Ajzen model and the restricted models.

Restrictions	Model	L(H0).	Λ	d.f.	$\chi_{0.95}^{2}$	Decision
None	Extended Ajzen Model	−324.48				
H0: MOA = SUC = 0	Ajzen Original Model	−343.72	38.48	1	3.84	Rejected
H0: SUC = 0	Ajzen Original Model with MOA	−325.70	2.44	1	3.84	Not rejected
H0: MOA = 0	Ajzen Original Model with SUC	−342.26	17.78	1	3.84	Rejected

**Table 6:** Testing the theoretical model by SEM (Moderator COO).

Variables	Ajzen Original Model with MOA				Ajzen Original Model			
	Basic Model		Moderation Model		Basic Model		Moderation Model	
	Std. β	*p*-Value	Std. β	*p*-Value	Std. β	*p*-Value	Std. β	*p*-Value
Direct effect								
ATT	0.67	0.00	0.75	0.00	0.75	0.00	0.85	0.00
INJ	0.21	0.00	0.09	0.65	0.32	0.00	0.24	0.18
PBC	0.05	0.20	−0.03	0.65	0.07	0.07	0.00	0.99
MOA	0.23	0.00	0.32	0.05				
ATT × PRS			−0.01	0.76			−0.02	0.70
INJ × PRS			0.02	0.52			0.01	0.75
PBC × PRS			0.04	0.06			0.04	0.05
MOA × PRS			−0.02	0.46				
Control effect								
COO	−0.03	0.34	−0.12	0.01	−0.01	0.85	−0.13	0.01
R2	0.98		0.98		0.98		0.98	
Correct R2	0.98		0.98		0.98		0.98	
*p*-value(F)	0.00		0.00		0.00		0.00	
Log-likelihood	−325.02	−318.39	−343.69	−334.98				
x2	382.98	6203.45	281.88	4727.43				
CFI	0.96	0.71	0.97	0.71				
TLI	0.95	0.66	0.96	0.649				
RMSEA	0.08	0.21	0.08	0.228				

**Table 7:** Testing the theoretical model by SEM (Moderator PRS).

Variables	Ajzen Original Model with MOA				Ajzen Original Model			
	Basic Model		Moderation Model		Basic Model		Moderation Model	
	Std. β	*p*-Value	Std. β	*p*-Value	Std. β	*p*-Value	Std. β	*p*-Value
Direct effect								
ATT	0.65	0.00	0.78	0.00	0.70	0.00	0.82	0.00
INJ	0.20	0.00	0.17	0.43	0.30	0.00	0.45	0.03
PBC	0.00	0.91	−0.31	0.00	0.01	0.72	−0.25	0.01
MOA	0.21	0.00	0.43	0.01				
ATT × PRS			−0.03	0.57			−0.03	0.57
INJ × PRS			0.00	0.94			−0.04	0.31
PBC × PRS			0.11	0.00			0.10	0.00
MOA × PRS			−0.06	0.09				
Control effect								
PRS	0.09	0.07	−0.03	0.54	0.13	0.01	−0.02	0.78
R2	0.98		0.98		0.98		0.98	
Correct R2	0.98		0.98		0.98		0.98	
*p*-value(F)	0.00		0.00		0.00		0.00	
Log-likelihood	−323.36	−309.34	−338.65	−324.44				
x2	517.14	8825.65	410.21	6.353.14				
CFI	0.95	0.65	0.95	0.66				
TLI	0.93	0.59	0.93	0.60				
RMSEA	0.08	0.24	0.09	0.25				

**Table 8:** Summary of the results relating to the three groups of hypotheses.

	Hypothesis	Result
H1a.	“Attitude” positively affects “consumption intention” of fish fed with insect meal.	Hypothesis confirmed
H1b.	“Perceived behavioral control” positively affects “consumption intention” of fish fed with insect meal.	Hypothesis rejected
H1c.	“Injunctive norms” positively affects “consumption intention” of fish fed with insect meal.	Hypothesis confirmed
H2a.	“Moral attitude” positively affects “consumption intention” of fish fed with insect meal.	Hypothesis confirmed
H2b.	“Sustainability consciousness” positively affects “consumption intention” of fish fed with insect meal.	Hypothesis rejected
H3a.	“Country of origin” of fish positively mediates the relationship between “attitude”, “perceived behavioral control”, “injunctive norms”, MOA, and “sustainability consciousness” and “consumption intention” of fish fed with insect meal.	Hypothesis rejected
H3b.	“Price sensitivity” positively mediates the relationship between “attitude”, “perceived behavioral control”, “injunctive norms”, “moral attitude”, and “sustainability consciousness” and “consumption intention” of fish fed with insect meal.	Hypothesis rejected

## Data Availability

The data that support the findings of this study are available from the corresponding author, upon reasonable request.

## Abbreviations

ATT	Attitude
AVE	Average Variance Extracted
CFI	Comparative fix index
COO	Country of origin
CR	Composite Reliability
FA	Factor loading analysis
FL	Factor Loadings
FM	Fish meal
IM	Insect meal
INJ	Injunctive norms
INT	Consumption intention of fish fed with IM
KMO	Kaiser-Meyer-Olkin
MOA	Moral attitude
MON	Moral norm
PBC	Perceived behavioral control
PRS	Price sensitivity
r	Pearson correlation coefficients
RMSEA	Root mean square error of approximation
SD	Standard deviation values
SEM	Structural equation model
SRMR	Standardized root mean squared residual
SUC	Sustainability consciousness
SUN	Subjective norm
TLI	Tucker–Lewis index
TPB	Theory of Planned Behavior

## Appendix A

### Survey Introduction

The increase in the world population, the increased awareness of the significant impact of eating habits on personal health, and the mental and physical health benefits of fish consumption have led to a notable increase in demand for seafood which will reach 28 million tons in 2030. This increase in demand can only be met through fish farming, i.e., aquaculture.

However, the main problem lies in feeding farmed fish, which are still mostly fed with fishmeal today. This entails significant negative impacts not only on the environment but also on the economic and social levels. The unsustainability of this practice has required an urgent rethinking of the protein sources used in fish farming (and beyond).

An alternative has been provided by the European Union, which has recently allowed the breeding of fish with feed derived from some insects.

Recent scientific research has shown that some insects possess numerous properties: the presence of a quantity of proteins, minerals, and vitamins very similar to that of fish meal, as well as high in energy, fat, and fiber; naturally present in the natural diet of freshwater and marine fish; the possibility of local production by small farmers; low environmental impact.

**Table A1:** Questionnaire items and their source of adoption.

Constructs	Questions of the Survey	Reference Bibliography
Attitude	Consuming fish fed with insect meal is a good thing.	[25,146]
	2. Consuming insects as an ingredient should be promoted in food production.	
	3. I feel positive about the idea of consuming fish fed with insect meal.	
Injunctive norms	1. Most people whose opinion I value would approve of my eating fish fed with insect meal.	[25,49]
	2. People I respect would consume fish fed with insect meal.	
	3. I would consume fish fed with insect meal if my friends recommended eating them.	
	4. I would consume fish fed with insect meal if dietary guidelines recommended eating them.	
Perceived behavior control	I believe that consuming fish fed with insect meal in the coming year is possible.	[25,49,147]
	2. Eating fish fed with insect meal in the next year is completely up to me.	
	3. I watch carefully what I eat.	
Consumption intention	My willingness to eat fish fed with insect meal is large.	[25,49,148]
	2. I intend to consume fish fed with insect meal when they are launched on the Italian markets.	
	3. The chance I will eat fish fed with insect meal in the next year is high.	
Country of origin	I think that fish reared in Italy is more reliable than those made in other countries.	[149]
	2. I think the quality of fish reared in Italy is better than those made in other countries.	
	3. I think that fish reared in Italy look better than those made in other countries” (i.e., they have superior packaging).	
Price sensitivity	It is acceptable to pay a premium for fish fed with insect meal than for fish fed with conventional feed.	[99,150]
	2. I am willing to pay more for fish fed with insect meal than for fish fed with conventional feed.	
	3. I would be willing to spend extra per week to buy more sustainable food.	
	4. The price of a product is a good indicator of its quality.	
Sustainability consciousness	When I consume food, I try to make environmentally friendly food choices.	[26,147,151]
	2. We who are living now should make sure that people in the future enjoy the same quality of life as we do today.	
	3. Humans must maintain a balance with nature to survive.	
Moral attitude	Consuming fish fed with insect meal instead of conventionally fed fish would feel like making a personal contribution to something better.	[62]
	2. Consuming fish fed with insect meal instead of conventionally fed fish would feel like the morally right thing.	
	3. Consuming fish fed with insect meal instead of conventionally fed fish would make me feel like a better person.	
